# Tale of two hearts: a TNNT2 hypertrophic cardiomyopathy case report

**DOI:** 10.3389/fcvm.2023.1167256

**Published:** 2023-04-27

**Authors:** Justin H. Pham, John R. Giudicessi, Marysia S. Tweet, Lauren Boucher, D. Brian Newman, Jeffrey B. Geske

**Affiliations:** ^1^ Mayo Clinic Alix School of Medicine, Mayo Clinic, Rochester, MN, United States; ^2^ Department of Cardiovascular Medicine, Mayo Clinic, Rochester MN, United States; ^3^ Department of Molecular Pharmacology and Experimental Therapeutics, Mayo Clinic, Rochester, MN, United States

**Keywords:** hypertrophic cardiomyopathy, TNNT2, cardiac troponin T, familial cardiomyopathy, incomplete genetic penetrance, variable genetic expressivity, case report

## Abstract

Hypertrophic cardiomyopathy (HCM) is a heritable cardiomyopathy that is predominantly caused by pathogenic mutations in sarcomeric proteins. Here we report two individuals, a mother and her daughter, both heterozygous carriers of the same HCM-causing mutation in cardiac Troponin T (*TNNT2*). Despite sharing an identical pathogenic variant, the two individuals had very different manifestations of the disease. While one patient presented with sudden cardiac death, recurrent tachyarrhythmia, and findings of massive left ventricular hypertrophy, the other patient manifested with extensive abnormal myocardial delayed enhancement despite normal ventricular wall thickness and has remained relatively asymptomatic. Recognition of the marked incomplete penetrance and variable expressivity possible in a single *TNNT2*-positive family has potential to guide HCM patient care.

## Introduction

Hypertrophic cardiomyopathy (HCM) is characterized by left ventricular hypertrophy (LVH) in the absence of other explanatory causes such as systemic hypertension, valvular disease, or infiltrative disease. In clinical practice, HCM is diagnosed through noninvasive cardiac imaging, typically echocardiography or magnetic resonance imaging (MRI), and defined by unexplained left ventricular wall thickness ≥15 mm in adults, or ≥13 mm in adults who have a first-degree relative with HCM (1).

HCM is believed to affect 1 out of every 500 adults in the general population, with the majority of cases being familial, following an autosomal dominant pattern of inheritance (2–4). The genetic basis of HCM is heterogenous, with over 1,400 disease-causing variants identified that have varying effects on a wide array of sarcomeric and Z-disc proteins in cardiomyocytes (5). Pathogenic alterations of myofilament proteins account for the majority of HCM cases and are associated with a high degree of phenotypic heterogeneity (6).

Approximately 4% of HCM cases are attributable to pathogenic alterations of the myofilament protein cardiac troponin T (1). Cardiac troponin T is a regulatory protein that functions as the tropomyosin-binding subunit of the thin filament troponin complex in the sarcomere and is encoded by the gene *TNNT2* (7, 8). As such, it plays an important role within myofilaments and is necessary for normal contractile function within cardiomyocytes. Over 30 distinct pathogenic/likely pathogenic variants affecting *TNNT2* gene products have been associated with HCM, with individual mutations being unique and private to individual families (3).

In the present study we report two individuals with HCM, a mother and her daughter, who both carry the same familial *TNNT2* mutation. Despite their genetic background, they presented with very different phenotypic manifestations of HCM, spanning clinical presentation, symptomatology, and cardiac imaging. This variable intra-familial phenotypic expressivity highlights the importance of genetic testing as a means of identifying susceptible individuals in families that carry HCM-causative genetic variants, especially considering most of the pathogenic mutations that cause HCM do not reliably predict clinical presentation, disease course, or prognosis (9–11). Furthermore, because of the vast phenotypic heterogeneity associated with HCM, many mutation carriers do not present with the typical finding of LVH and may be asymptomatic, going undiagnosed (9, 10).

## Family genetic history

This report focuses on two individuals of Caucasian descent, a woman presenting at age 18 (Patient A) and her mother (Patient B). Both were found to have a c.275 G > A mutation (p.Arg92Gln-TNNT2) in the gene *TNNT2* through genetic testing (GeneDx Laboratories, Gaithersburg, MD). No additional HCM-associated gene variants were reported in either patient.

**Figure 1:**
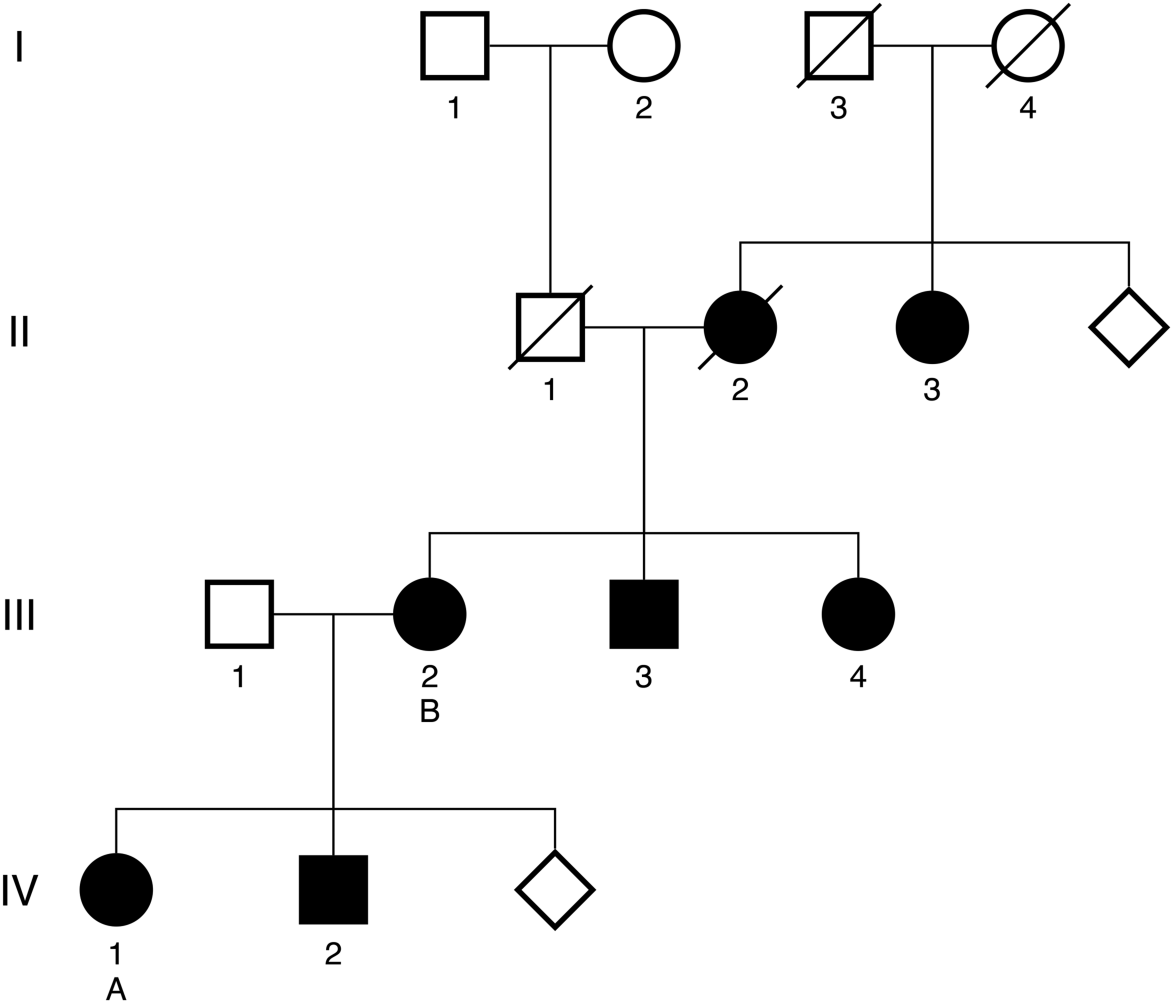
Targeted family pedigree. Family members with a confirmed diagnosis of HCM are denoted by solid dark shapes. Patient A and patient B are denoted by the letters A and B, respectively. Deceased family members are denoted with a diagonal line. Diamonds represent the presence of additional family members within a generation who have not been diagnosed with HCM.

Family pedigree is shown as Figure 1. Of note, five additional family members are known to have HCM, including one of patient A's brothers (IV-2), both of patient B's siblings (III-3, III-IV), patient B's mother (II-2), and one of patient B's maternal aunts (II-3). These additional family members have not yet undergone genetic testing with confirmed results. At present, patients A and B are the only confirmed carriers of the *TNNT2* variant in this family.

## Case presentation of patient A

**Figure 2:**
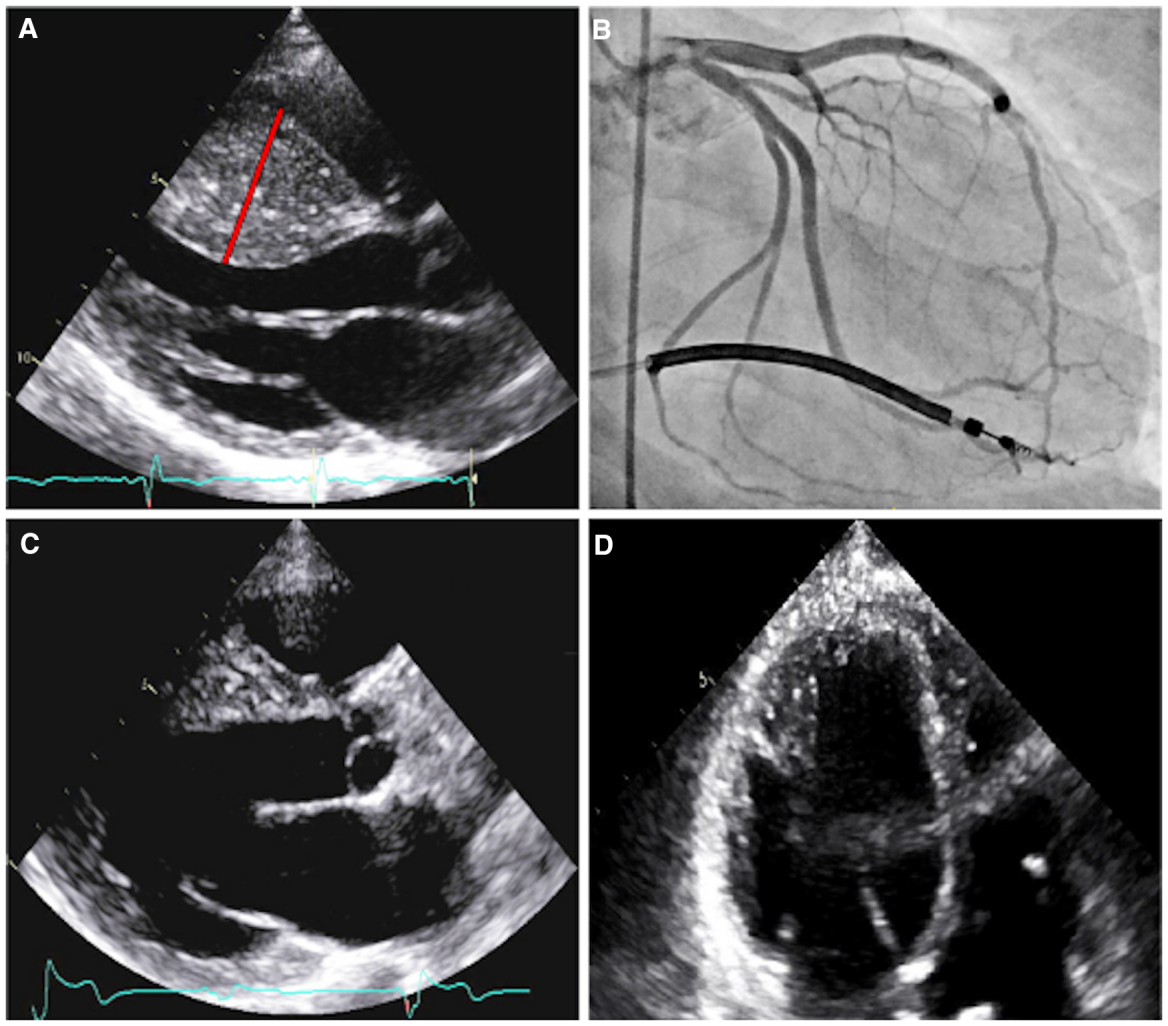
Imaging of patient A. Parasternal long axis echocardiogram prior to septal myectomy (**A**). The interventricular septum has reverse curve morphology, with a septal thickness measurement of 33 mm (red line) during diastole and a patent left ventricular outflow tract. Right anterior oblique caudal angiogram with prominent myocardial bridging of the large first and second septal perforators (**B**). Parasternal long axis echocardiogram, status-post septal myectomy (**C**). Four-chamber echocardiogram, with increased apical cavity size following septal myectomy (**D**).

Patient A was initially referred for cardiac evaluation when she experienced a sudden out-of-hospital cardiac arrest at age 18. She was swing dancing with friends when she suddenly lost consciousness and was found to be pulseless. Bystander cardiopulmonary resuscitation was performed for eight minutes. On-site automatic external defibrillator advised no shock. She regained full neurological function during hospitalization with a hypothermic protocol. Serial 12-lead electrocardiograms (ECGs) demonstrated left ventricular hypertrophy with ST-T depression in the anterolateral leads, with an initial QTc interval of 510 msec that normalized throughout the course of hospitalization. Transthoracic echocardiography revealed HCM with massively hypertrophied left ventricular septum (33 mm) with a reverse curve septal contour (Figure 2A). There was no outflow tract obstruction at rest or with Valsalva maneuver. She underwent single-lead implantable cardioverter-defibrillation (ICD) placement and was started on metoprolol succinate 25 mg daily.

ECG treadmill stress testing was performed two months following the initial hospitalization, which demonstrated a blunted blood pressure response from 80/58 mmHg to 90/46 mmHg and a peak VO_2_ of 25.3 ml/kg/min (66% of predicted). She then experienced three separate ambulation-associated syncopal episodes in the following months, all with appropriate ICD discharges. ICD interrogation revealed sinus tachycardia at 140–150 bpm preceding ventricular tachycardia that quickly degenerated into ventricular fibrillation, with rates above 300 bpm by the time the shocks were delivered. She was started on amiodarone at 400 mg per day in addition to her metoprolol therapy.

Following these events, she underwent repeat ECG treadmill stress testing and developed polymorphic ventricular tachycardia 4 min and 20 s into exercise. Her ICD did not discharge due to rates below the detection zone (detection zone 290 to 310 msec, whereas the event was at 280 msec). Manual chest compressions and 150 mg of IV amiodarone were administered with resultant return to sinus rhythm. She was directly admitted and electrophysiology (EP) study was performed, but no clinically inducible arrythmia could be found. The ICD was reprogrammed to detect ventricular tachycardia at a lower threshold, and she was transitioned to monotherapy with sotalol (80 mg twice daily). Thorascopic sympathectomy of the left sympathetic chain (T1–T4) was performed. During a subsequent ECG treadmill stress test she developed global ST depression with ventricular tachycardia approximately four minutes into exercise, followed by ventricular fibrillation and loss of consciousness with ICD discharge. She regained a normal sinus rhythm and consciousness shortly thereafter. Sotalol was discontinued and she was started on flecainide (100 mg twice daily).

Coronary angiography revealed marked myocardial bridging of large septal perforators (Figure 2B and Supplementary Video S1). She ultimately underwent coronary artery unroofing and extended septal myectomy through combined transaortic and transapical approaches to improve diastolic function and debulk her arrhythmogenic substrate (Figures 2C, D). Pathology demonstrated histological features consistent with HCM, including moderate to marked myocyte hypertrophy, mild to focally moderate myocyte disarray, and mild to focally moderate interstitial fibrosis. Flecainide was discontinued and sotalol (120 mg twice daily) was resumed on post-operative day four.

A follow up EP study was pursued four months post-operatively for ventricular arrythmia substrate modification. Substrate mapping was performed in the right and left ventricles with endocardial radiofrequency ablation of fragmented potentials in the right ventricular septum, left ventricular septum, and lateral mitral annular region. She continued sotalol and did not have any peri-procedural ventricular tachyarrhythmias. Five months later her ICD discharged once more, though this was determined to be an inappropriate shock. Atrial fibrillation with a rapid ventricular response was suspected. She was asymptomatic during the episode aside from mild chest discomfort. The ICD detection zone was increased to 205–210 bpm, which has prevented further inappropriate discharges.

One year after the ablation, she sustained an occlusion of the right posterior cerebral artery at the P2–P3 segment, suggestive of a cardioembolic source. A transesophageal echocardiogram revealed a highly mobile mass on the pacemaker lead in the high right atrium but no left heart masses. There was no intracardiac shunt to facilitate paradoxical embolism. It was ultimately thought that a thrombus could have formed within the left ventricular apex, where apical dyskinesia could predispose to thrombus formation. She was started on warfarin (5 mg daily) with bridging enoxaparin and monitored with a goal INR of 2–3. She experienced a transient ischemic attack approximately three years later when her INR was subtherapeutic (1.8). Her INR goal was empirically increased to 2.5–3.5.

Given the development of lower extremity edema and worsening diastolic function, she was placed on furosemide 20 mg daily and was transitioned to a cardiac resynchronization therapy device. She remained hemodynamically stable and following diuresis, right heart catheterization demonstrated normal right atrial pressure (6 mmHg), right ventricular systolic pressure (21 mmHg), mean pulmonary artery pressure (14 mmHg), and pulmonary capillary wedge pressure (12 mmHg). Serial echocardiographic surveillance has demonstrated preserved left ventricular systolic function, with stable low-normal ejection fraction of 54%. Her functional capacity has remained reduced but stable, with peak exercise capacity ranging between 31% and 39% of predicted, and peak VO_2_ of 12.7 (36% of predicted).

## Case presentation of patient B

**Figure 3:**
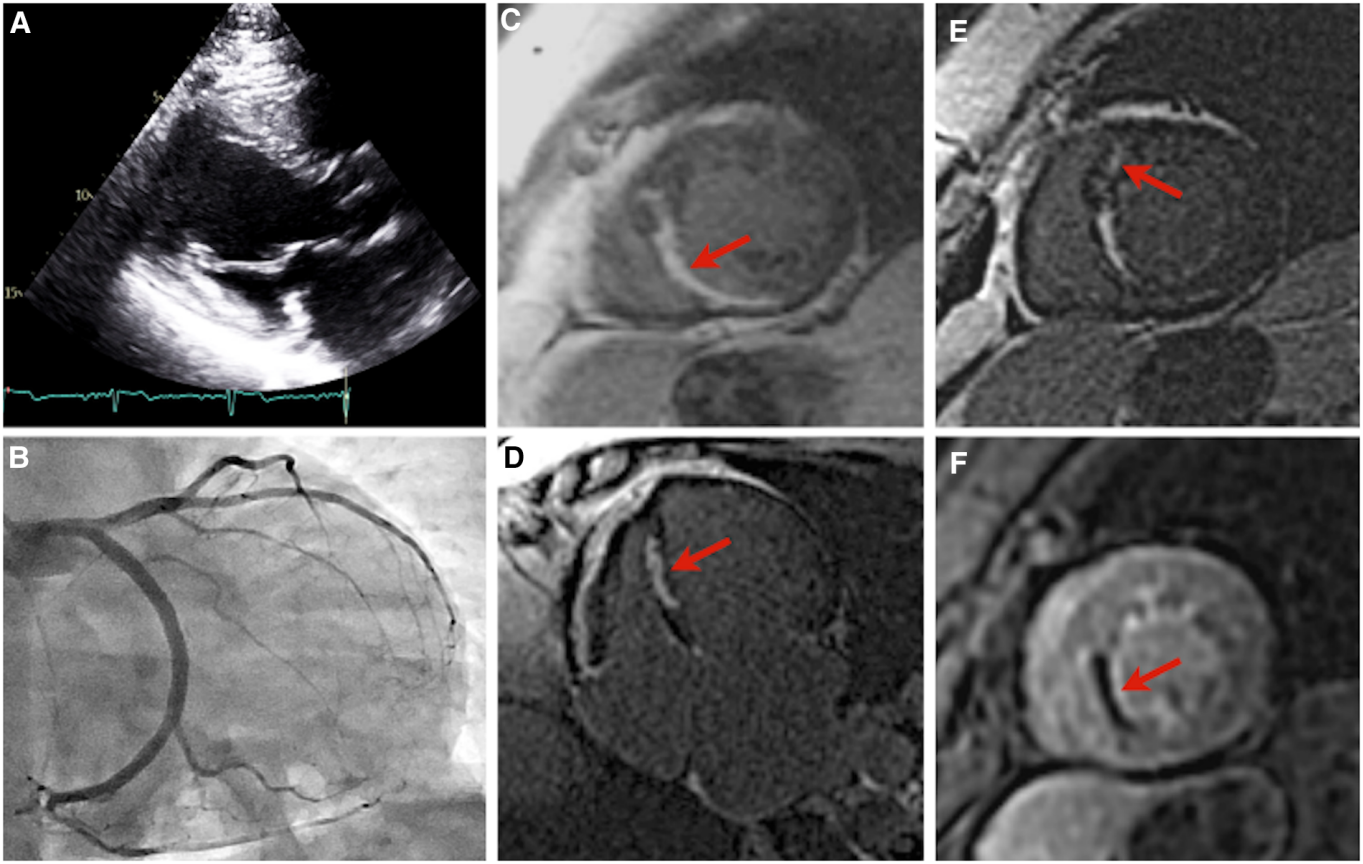
Imaging of patient B. Parasternal long axis echocardiogram, with an interventricular septal thickness measurement of 11 mm during diastole, and a patent left ventricular outflow tract (**A**). Right anterior oblique caudal angiogram demonstrating a diminutive left anterior descending artery (**B**). Images (**C–F**): cardiac magnetic resonance imaging of patient B. Near-transmural delayed gadolinium enhancement of the mid inferior and inferoseptal wall segments, short-axis view (**C**). Near-transmural delayed gadolinium enhancement of the mid inferior and inferoseptal wall segments, four-chamber view (**D**). Focus of mid-myocardial delayed gadolinium enhancement in the mid anteroseptal wall segment, short-axis view (**E**). Near-transmural septal hypoperfusion, short-axis view (**F**).

Patient B was first referred at age 46 for genetic evaluation shortly after patient A began workup for HCM, as per familial screening guidelines, and was revealed to be a carrier of the pathogenic *TNNT2* variant. Her initial transthoracic echocardiogram revealed a maximal wall thickness of 11 mm with hypokinesis in the basal to mid inferior wall and basal to mid anteroseptal walls, with no evidence of obstruction (Figure 3A). ECG demonstrated baseline ST and T wave abnormalities. Prior to this evaluation she had experienced chronic episodic chest pain without other cardiac symptoms or clear precipitating factors, though cardiac diagnostic evaluation had never been pursued. Holter monitoring revealed a 7-beat run of nonsustained ventricular tachycardia at 129 bpm. A treadmill stress echocardiogram with maximal exertion resulted in no symptoms of chest pain. Electrocardiographic findings were within normal limits aside from baseline ST and T wave abnormalities. There was normal augmentation of left ventricular function with an increase in ejection fraction from 55% at rest to 65% during peak stress. No new regional wall motion abnormalities were identified. No further coronary evaluation was pursued.

She remained well from a cardiovascular standpoint until 8 years thereafter, when she presented to an outside emergency department with palpitations and intermittent chest discomfort. Newly identified atrial tachycardia was present with a ventricular rate of 130–150 bpm. She developed significant bradycardia (30 s bpm) with first-degree AV block after 3.5 mg of intravenous metoprolol tartrate. Her troponin I level was also noted to be mildly elevated to 1.352 ng/ml (reference range ≤0.013 ng/ml) and NT-proBNP to 260 pg/ml (reference ≤100 pg/ml). Coronary angiography revealed normal variant anatomy with a diminutive LAD (Figure 3B). She was monitored overnight with no further tachyarrhythmia or chest discomfort and discharged on dual anti-platelet therapy and high-intensity statin therapy.

Subsequent outpatient cardiac MRI demonstrated wall thinning and akinesia of the mid inferior and inferior septal segments with near transmural delayed enhancement hypoperfusion, and focal myocardial delayed enhancement of the mid anterior septal wall segment (Figures 3C–F). Repeat transthoracic echocardiography demonstrated a severely enlarged left atrium, left ventricular ejection fraction 54%, and ventricular septal thickness of 11 mm. Ambulatory Holter monitoring thereafter demonstrated low ectopic burden with self-limiting runs of atrial tachycardia and two 4-beat runs of nonsustained ventricular tachycardia.

Overall, she has remained NYHA class I on medical therapy with metoprolol succinate (25 mg daily) and is scheduled to undergo ICD implantation in the future given her family history, scarring on cardiac MRI, and non-sustained ventricular tachycardia. Her troponin T level has remained stably elevated on serial outpatient measurements (19–20 ng/dl, reference range ≤10 ng/L).

## Discussion

Not all carriers of *TNNT2* mutations will present with HCM. Furthermore, pathogenic alterations to cardiac troponin T often present with marked incomplete genetic penetrance and variable expressivity (6). Previous cases have been reported in which *TNNT2* mutations are associated with only minor or subclinical left ventricular hypertrophy but carry a high risk of arrhythmia (12). *TNNT2* mutations have additionally been implicated in other myocardial diseases including dilated cardiomyopathy, restrictive cardiomyopathy, and left ventricular noncompaction (13–15). The profound variability in clinical presentation and patient outcomes associated with *TNNT2* mutations can complicate the diagnosis and treatment of the diseases they cause.

Patient A carried a high disease burden with treatment-resistant ventricular arrhythmia, even following coronary artery unroofing, septal myectomy, and left sympathectomy. It is possible that myocardial ischemia may have contributed to her arrhythmia, particularly in the case of her second treadmill stress test; however, she did not describe angina and no definite ischemic findings were present on cardiac imaging. Ranolazine has recently shown promise in reducing ventricular arrhythmic burden in select patients with HCM and could have been considered (16). Given her borderline ejection fraction, worsening diastolic function, and reduced functional capacity, close cardiovascular clinic follow-up of patient A is needed, with cardiac transplantation a potential future outcome.

Patient B had milder LV hypertrophy with evidence of microvascular angina. Overall, her presentation was more consistent with the typical phenotype seen in thin-filament HCM (17, 18). She had no functional limitations in her daily life and remained relatively asymptomatic until presenting to the emergency department with palpitations. Serial lab work in the outpatient setting revealed a chronically elevated baseline troponin level, consistent with HCM (19). Together, these findings suggest HCM rather than an acute infarct as the cause of her initial presentation. The pattern of patchy mid-wall late gadolinium enhancement involving the interventricular septum observed on her MR imaging (Figures 3C–E) is attributable to myocardial fibrosis and occurs in approximately 60% of HCM patients with LVH (20). A similar distribution of late gadolinium enhancement was previously documented in another p.Arg92Gln-TNNT2-positive individual who presented without symptoms or echocardiographic evidence of HCM (21). The functional implications of late gadolinium enhancement in the setting of HCM remain unclear. Previous studies have found it to be inconsequential, whereas others have demonstrated associations with increased myocardial stiffness, regional wall motion abnormalities, and diminished LV systolic function (20). Regions of fibrosis may also serve as arrhythmogenic substrates and the extent of scarring has been correlated with sudden cardiac death and major adverse cardiovascular events (21, 22). Because of this, the 2020 ACC/AHA guidelines have highlighted the clinical utility of late gadolinium enhancement evaluation in HCM sudden cardiac death risk stratification (23).

The genetic locus of *TNNT2* is 1q32.1 and its transcript contains 17 exons (11). The p.Arg92 codon within this gene has been described as a “hot spot” for mutation, and an p.Arg92Gln-TNNT2 pathogenic variant was determined to be the genetic culprit for the family in the current study. Other documented TNNT2 HCM-causative pathogenic/likely pathogenic variants include p.Arg278Cys-TNNT2, p.Arg92Leu-TNNT2, p.Arg92Trp-TNNT2, p.*Δ*Glu163-TNNT2, p.Ala104Val-TNNT2, and p.Arg278His-TNNT2 (24). Pathogenic variants of *TNNT2* gene product variants account for a relatively small subset of HCM cases compared to other myofilament genes such as *MYBPC3* and *MYH7* (2, 25). Though both patients in this report presented with nonobstructive HCM, it should be noted that both obstructive and nonobstructive phenotypes can arise from *TNNT2* mutations (24).

A growing body of work in the past few years has shown that the high degree of phenotypic heterogeneity observed in HCM may be due, at least in part, to the additive effects of pro-hypertrophic common genetic variants across the genome. Recent genome-wide association studies have jointly identified over a dozen common (minor allele frequency >0.01) genetic variants that contribute to HCM risk and severity (26, 27). These common variants are distinct from the rarer sarcomeric genes (e.g., *TNNT2*) traditionally associated with HCM, but have been correlated with increased HCM risk in both carriers and noncarriers of pathogenic sarcomeric protein variants (27). Polygenic risk score metrics have been constructed to calculate the additive genetic effects that these common variants have on an individual's HCM risk. Importantly, an upper quintile PRS has been linked to a relative increase in disease severity, measured through adverse clinical events and degree of LVH, while a lower score has protective effects (26). Thus, interindividual differences in “genetic background” may contribute to differences in expressivity between two carriers of the same pathogenic *TNNT2* variant. Additionally, modifiable risk factors such as hypertension have similarly been correlated with increased disease risk and severity in both carriers and noncarriers of pathogenic sarcomeric protein variants (27, 28). These findings suggest the potential benefit in assessing polygenic burden status and modifiable risk factors for HCM prognostication (28).

Genetic testing should be considered in any patient who fulfills diagnostic criteria for HCM, as genetic testing is the preferred method of family screening when a causative mutation is identified (23). Due to the genetic heterogeneity of HCM, a comprehensive panel should be used for screening that covers not only the main sarcomeric culprits (e.g., *TNNT2*, *MYH7*, *MYBPC3*, *TNNI3*, *ACTC*), but also other causes including RASopathies, mitochondrial proteins, and glycogen storage diseases (29). Although important for family screening, genetic analysis does not typically alter therapeutic decision-making in the management of HCM.

**Figure 4:**
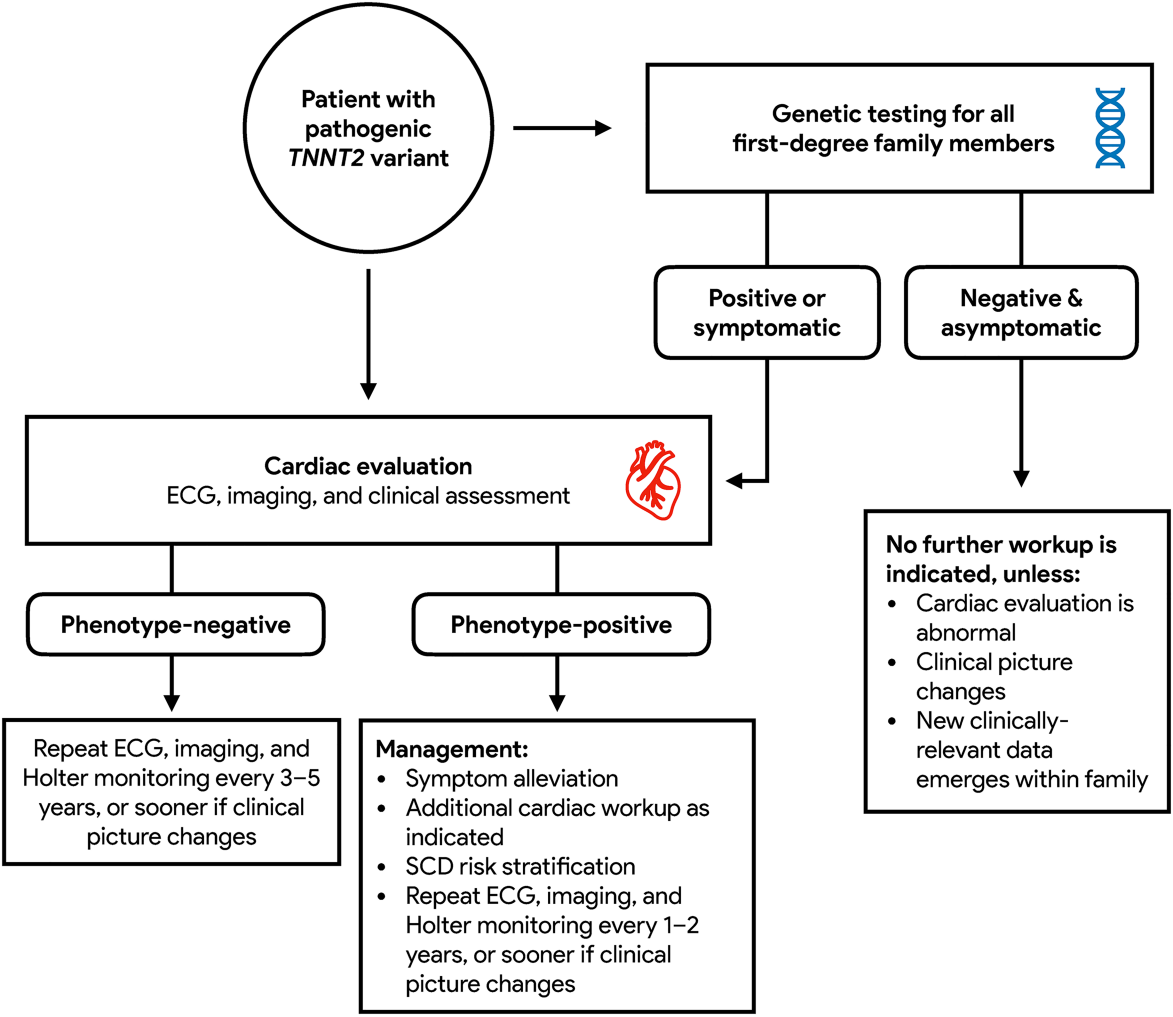
Recommended diagnostic paradigm for *TNNT2* HCM.

In general, patients who test positive for pathogenic variants of *TNNT2* are recommended to undergo cardiac evaluation due to their increased risk for the development of HCM (30). Mutation carriers who are symptomatic or phenotype-positive should continue ongoing monitoring inclusive of ECG, transthoracic echocardiography, and ambulatory Holter monitoring every 1–2 years (23). Risk assessment for sudden cardiac death, inclusive of stress testing and cardiac MRI should also be performed, with a low threshold for additional diagnostic cardiac workup as indicated. The frequency of follow up beyond general guidelines should be further tailored to individual patients on the basis of symptomatology, additional genetic results, functional capacity, changes in clinical status, and course of disease progression (31). For patients in whom there is concern for HCM but echocardiography is inconclusive, cardiac MRI should be performed for diagnostic clarification. Mutation carriers who are asymptomatic or phenotype-negative should repeat screening every 3–5 years (23). First-degree relatives of mutation carriers who undergo genetic testing and are found not to be mutation carriers themselves are still recommended to have cardiac evaluation done. If cardiac workup returns negative and these patients are asymptomatic, they may be discharged from further follow-up but advised to seek re-assessment in the event that their clinical picture changes or new clinically relevant data emerges within the family (9). These recommendations are summarized as Figure 4.

## Conclusion

Though they account for a relatively small subset of HCM cases, pathogenic variants in *TNNT2* exemplify the marked incomplete penetrance and variable phenotypic expressivity seen in genetic cardiomyopathies. As we have reported, a single genotype can give rise to a wide range of presentations, from mild symptoms to sudden cardiac death. The two patients presented herein had very different phenotypic expressions of the same p.Arg92Gln-TNNT2 pathogenic variant, manifesting at different stages in their lives. One individual carried a relatively high burden of HCM manifesting as massive left ventricular hypertrophy and recurrent tachyarrhythmia. The other had an overall milder presentation with subclinical to minimal left ventricular hypertrophy. This report adds to the ever-growing body of documentation that highlights the highly heterogenous phenotypes of *TNNT2* cardiomyopathies.

## Data Availability

The original contributions presented in the study are included in the article, further inquiries can be directed to the corresponding author.

## References

[1] Cirino AL Ho C Hypertrophic cardiomyopathy overview. In: Adam MP Everman DB Mirzaa GM Pagon RA Wallace SE Bean LJH, editors. Genereviews(®). Seattle, WA: University of Washington, Seattle. Copyright © 1993–2022, University of Washington, Seattle. GeneReviews is a registered trademark of the University of Washington, Seattle. All rights reserved. (1993). p. 1–6. 20301725

[2] Geske JB Ommen SR Gersh BJ. Hypertrophic cardiomyopathy: clinical update. JACC Heart Fail. (2018) 6(5):364–75. 10.1016/j.jchf.2018.02.010 29655825

[3] Richard P Villard E Charron P Isnard R. The genetic bases of cardiomyopathies. J Am Coll Cardiol. (2006) 48(9, Supplement):A79–A89. 10.1016/j.jacc.2006.09.014

[4] Maron BJ. Hypertrophic cardiomyopathy: a systematic review. JAMA. (2002) 287(10):1308–20. 10.1001/jama.287.10.1308 11886323

[5] Maron BJ Maron MS. Hypertrophic cardiomyopathy. Lancet. (2013) 381(9862):242–55. 10.1016/S0140-6736(12)60397-3 22874472

[6] Marian AJ. Molecular genetic basis of hypertrophic cardiomyopathy. Circ Res. (2021) 128(10):1533–53. 10.1161/CIRCRESAHA.121.318346 33983830 PMC8127615

[7] Townsend PJ Farza H MacGeoch C Spurr NK Wade R Gahlmann R Human cardiac troponin T: identification of fetal isoforms and assignment of the TNNT2 locus to chromosome 1q. Genomics. (1994) 21(2):311–6. 10.1006/geno.1994.1271 8088824

[8] Wei B Jin JP. TNNT1, TNNT2, AND TNNT3: isoform genes, regulation, and structure-function relationships. Gene. (2016) 582(1):1–13. 10.1016/j.gene.2016.01.006 26774798 PMC5325693

[9] Elliott PM Anastasakis A Borger MA Borggrefe M Cecchi F Charron P 2014 Esc guidelines on diagnosis and management of hypertrophic cardiomyopathy: the task force for the diagnosis and management of hypertrophic cardiomyopathy of the European society of cardiology (ESC). Eur Heart J. (2014) 35(39):2733–79. 10.1093/eurheartj/ehu284 25173338

[10] Landstrom AP Ackerman MJ. Mutation type is not clinically useful in predicting prognosis in hypertrophic cardiomyopathy. Circulation. (2010) 122(23):2441–9; discussion 50. 10.1161/CIRCULATIONAHA.110.954446 21135372 PMC6309993

[11] Gao G Liu G Chen W Tong Y Mao C Liu J A novel nonsense mutation in Tnnt2 in a Chinese pedigree with hypertrophic cardiomyopathy: a case report. Medicine (Baltimore). (2020) 99(34):e21843. 10.1097/MD.0000000000021843 32846832 PMC7447477

[12] Teekakirikul P Kelly MA Rehm HL Lakdawala NK Funke BH. Inherited cardiomyopathies: molecular genetics and clinical genetic testing in the postgenomic era. J Mol Diagn. (2013) 15(2):158–70. 10.1016/j.jmoldx.2012.09.002 23274168

[13] Hershberger RE Pinto JR Parks SB Kushner JD Li D Ludwigsen S Clinical and functional characterization of Tnnt2 mutations identified in patients with dilated cardiomyopathy. Circ Cardiovasc Genet. (2009) 2(4):306–13. 10.1161/CIRCGENETICS.108.846733 20031601 PMC2900844

[14] Parvatiyar MS Pinto JR Dweck D Potter JD. Cardiac troponin mutations and restrictive cardiomyopathy. J Biomed Biotechnol. (2010) 2010:350706. 10.1155/2010/350706 20617149 PMC2896668

[15] Luedde M Ehlermann P Weichenhan D Will R Zeller R Rupp S Severe familial left ventricular non-compaction cardiomyopathy due to a novel troponin T (Tnnt2) mutation. Cardiovasc Res. (2010) 86(3):452–60. 10.1093/cvr/cvq009 20083571

[16] Argirò A Zampieri M Dei LL Ferrantini C Marchi A Tomberli A Safety and efficacy of ranolazine in hypertrophic cardiomyopathy: real-world experience in a national referral center. Int J Cardiol. (2023) 370:271–8. 10.1016/j.ijcard.2022.10.014 36228766

[17] Coppini R Ho CY Ashley E Day S Ferrantini C Girolami F Clinical phenotype and outcome of hypertrophic cardiomyopathy associated with thin-filament gene mutations. J Am Coll Cardiol. (2014) 64(24):2589–600. 10.1016/j.jacc.2014.09.059 25524337 PMC4270453

[18] Watkins H McKenna WJ Thierfelder L Suk HJ Anan R O’Donoghue A Mutations in the genes for cardiac troponin T and alpha-tropomyosin in hypertrophic cardiomyopathy. N Engl J Med. (1995) 332(16):1058–64. 10.1056/NEJM199504203321603 7898523

[19] Burczak DR Newman DB Jaffe AS Ackerman MJ Ommen SR Geske JB. High-Sensitivity cardiac troponin T elevation in hypertrophic cardiomyopathy Is associated with ventricular arrhythmias. Mayo Clin Proc. (2023) 98(3):410–8. 10.1016/j.mayocp.2022.08.010 36868748

[20] Noureldin RA Liu S Nacif MS Judge DP Halushka MK Abraham TP The diagnosis of hypertrophic cardiomyopathy by cardiovascular magnetic resonance. J Cardiovasc Magn Reson. (2012) 14(1):17. 10.1186/1532-429X-14-17 22348519 PMC3309929

[21] Strijack B Ariyarajah V Soni R Jassal DS Greenberg CR McGregor R Late gadolinium enhancement cardiovascular magnetic resonance in genotyped hypertrophic cardiomyopathy with normal phenotype. J Cardiovasc Magn Reson. (2008) 10(1):58. 10.1186/1532-429X-10-58 19087273 PMC2633334

[22] Moon JC McKenna WJ McCrohon JA Elliott PM Smith GC Pennell DJ. Toward clinical risk assessment in hypertrophic cardiomyopathy with gadolinium cardiovascular magnetic resonance. J Am Coll Cardiol. (2003) 41(9):1561–7. 10.1016/S0735-1097(03)00189-X 12742298

[23] Ommen SR Mital S Burke MA Day SM Deswal A Elliott P 2020 Aha/Acc guideline for the diagnosis and treatment of patients with hypertrophic cardiomyopathy: a report of the American college of cardiology/American heart association joint committee on clinical practice guidelines. Circulation. (2020) 142(25):e558–e631. 10.1161/cir.0000000000000937 33215931

[24] Pasquale F Syrris P Kaski JP Mogensen J McKenna WJ Elliott P. Long-term outcomes in hypertrophic cardiomyopathy caused by mutations in the cardiac troponin T gene. Circ Cardiovasc Genet. (2012) 5(1):10–7. 10.1161/CIRCGENETICS.111.959973 22144547

[25] Lorenzini M Norrish G Field E Ochoa JP Cicerchia M Akhtar MM Penetrance of hypertrophic cardiomyopathy in sarcomere protein mutation carriers. J Am Coll Cardiol. (2020) 76(5):550–9. 10.1016/j.jacc.2020.06.011 32731933 PMC7397507

[26] Tadros R Francis C Xu X Vermeer AMC Harper AR Huurman R Shared genetic pathways contribute to risk of hypertrophic and dilated cardiomyopathies with opposite directions of effect. Nat Genet. (2021) 53(2):128–34. 10.1038/s41588-020-00762-2 33495596 PMC7611259

[27] Harper AR Goel A Grace C Thomson KL Petersen SE Xu X Common genetic variants and modifiable risk factors underpin hypertrophic cardiomyopathy susceptibility and expressivity. Nat Genet. (2021) 53(2):135–42. 10.1038/s41588-020-00764-0 33495597 PMC8240954

[28] Biddinger KJ Jurgens SJ Maamari D Gaziano L Choi SH Morrill VN Rare and common genetic variation underlying the risk of hypertrophic cardiomyopathy in a national biobank. JAMA Cardiol. (2022) 7(7):715–22. 10.1001/jamacardio.2022.1061 35583889 PMC9118016

[29] Monserrat L. Perspectives on current recommendations for genetic testing in HCM. Glob Cardiol Sci Pract. (2018) 2018(3):23. 10.21542/gcsp.2018.23 30393635 PMC6209450

[30] Hershberger RE Lindenfeld J Mestroni L Seidman CE Taylor MR Towbin JA. Genetic evaluation of cardiomyopathy–a heart failure society of America practice guideline. J Card Fail. (2009) 15(2):83–97. 10.1016/j.cardfail.2009.01.006 19254666

[31] Heidenreich PA Bozkurt B Aguilar D Allen LA Byun JJ Colvin MM 2022 AHA/ACC/HFSA guideline for the management of heart failure: a report of the American College of Cardiology/American Heart Association Joint Committee on clinical practice guidelines. Circulation. (2022) 145(18):e895–e1032. 10.1161/CIR.0000000000001063 35363499

